# UNDERSTANDING UNMET NEEDS AND SUPPORT AMONG DIVERSE FAMILY CAREGIVERS ENGAGED IN COMPLEX

**DOI:** 10.1093/geroni/igae098.0772

**Published:** 2024-12-31

**Authors:** Janice Bell, Robin Whitney, Benjamin Link, Shikha Bhurtel, Orly Tonkikh, Jessica Famula, Kathleen Kelly, Heather Young

**Affiliations:** UC Davis, Davis, California, United States; San Jose State, San Jose, California, United States; University of California Davis, Davis, California, United States; UC Davis, Davis, California, United States; University of California Davis, Davis, California, United States; University of California Davis, Davis, California, United States; Family Caregiving Alliance, San Francisco, California, United States; UC Davis, Ashland, Oregon, United States

## Abstract

More than 56 million family caregivers provided unpaid care to a family member in the last year. This group is increasing in number and diversity, while providing more intense and complex care, as more older adults are living longer with chronic and serious illness. We used assessment and service data from a large statewide database (CareNavTM) collected by 11 California Caregiver Resources Centers statewide to describe the caregiving demands, unmet needs and resource gaps of diverse family caregivers seeking help from the centers. Almost half of the caregivers identified as Black non-Hispanic, Hispanic/Latino, Asian American/Pacific Islander, Native American/Alaska Native or multi-racial/other. Older caregivers and those with low income were more likely to provide ≥40 hours of care weekly and to provide high intensity care (measured with the number of personal care activities supported and weekly hours of care). Younger caregivers, those in racial/ethnic groups other than White non-Hispanic, and those with low income were more likely to perform medical/nursing tasks. These groups of caregivers were also the least likely to have paid or unpaid help and the most likely to report adverse outcomes including fair or poor health status, worsening health and moderate or severe depressive symptoms. Taken together, these findings inform the California Department ofn Aging efforts to develop an equity plan for caregivers and are useful to other states seeking to develop policy to assure that all family caregivers have a range of high-quality services and supports that promote their physical, emotional, and financial wellbeing.

